# Bioinformatics analysis reveals novel hub gene pathways associated with IgA nephropathy

**DOI:** 10.1186/s40001-020-00441-2

**Published:** 2020-09-07

**Authors:** Xue Jiang, Zhijie Xu, Yuanyuan Du, Hongyu Chen

**Affiliations:** 1 Department of Nephropathy, Hangzhou Hospital of Traditional Chinese Medicine, Hangzhou, 310012 Zhejiang China; 2 Department of Urology, The First Affiliated Hospital School of Medicine, Zhejiang University, Hangzhou, 310009 Zhejiang China

**Keywords:** IgA nephropathy, Gene expression profiling, Bioinformatics analysis

## Abstract

**Background:**

Immunoglobulin A nephropathy (IgAN) is the most common primary glomerulopathy worldwide. However, the molecular events underlying IgAN remain to be fully elucidated. This study aimed to identify novel biomarkers of IgAN through bioinformatics analysis and elucidate the possible molecular mechanism.

**Methods:**

Based on the microarray datasets GSE93798 and GSE37460 downloaded from the Gene Expression Omnibus database, the differentially expressed genes (DEGs) between IgAN samples and normal controls were identified. Using the DEGs, we further performed a series of functional enrichment analyses. Protein–protein interaction (PPI) networks of the DEGs were constructed using the STRING online search tool and were visualized using Cytoscape. Next, hub genes were identified and the most important module among the DEGs, Biological Networks Gene Ontology tool (BiNGO), was used to elucidate the molecular mechanism of IgAN.

**Results:**

In total, 148 DEGs were identified, comprising 53 upregulated genes and 95 downregulated genes. Gene Ontology (GO) analysis indicated that the DEGs for IgAN were mainly enriched in extracellular exosome, region and space, fibroblast growth factor stimulus, inflammatory response, and innate immunity. Module analysis showed that genes in the top 1 significant module of the PPI network were mainly associated with innate immune response, integrin-mediated signaling pathway and inflammatory response. The top 10 hub genes were constructed in the PPI network, which could well distinguish the IgAN and control group in monocyte and tissue samples. We finally identified the integrin subunit beta 2 (ITGB2) and Fc fragment of IgE receptor Ig (FCER1G) genes that may play important roles in the development of IgAN.

**Conclusions:**

We identified key genes along with the pathways that were most closely related to IgAN initiation and progression. Our results provide a more detailed molecular mechanism for the development of IgAN and novel candidate gene targets of IgAN.

## Background

IgA nephropathy (IgAN), the most prevalent type of glomerulonephritis in humans, is characterized by mesangial cell proliferation, the expansion of the glomerular mesangial matrix. Nearly 25–30% of affected patients develop end-stage renal disease. Presently, several clinical biomarkers have been identified to be associated with IgAN progression, such as proteinuria, serum creatinine, hypertension and advanced histological involvement [1]. In 2011 [2], Suzuki et al. hypothesized that the pathogenesis of IgAN is based on four hits: first, the occurrence of an abnormal IgA1 glycosylation process leading to galactose-deficient IgA1 (Gd-IgA1); second, the formation of antiglycan antibodies against Gd-IgA1; third, the formation of nephrogenic circulating immune complexes; fourth, the deposition of these complexes in the mesangium of glomeruli, leading to renal injury with variable clinical expression. However, the exact pathogenesis is not very clear.

Many studies have also shown a genetic predisposition to IgAN [3]. Serino et al. found six significantly upregulated miRNAs, two of which modulate the O-glycosylation process of IgA1. Specifically, let-7b regulates the gene GALNT2 and miR-148 modulates the gene target C1GALT1, which has been considered an underlying biomarker to predict the probability of IgAN [4, 5]. Wang et al. found that low urinary levels of miR-29b and miR-29c are correlated with proteinuria and renal function. High levels of miR-93 were correlated with glomerular scarring. miR-200a, miR-200b, and miR-429 have also been suggested as potential biomarkers to monitor the progression of the disease at the renal level in IgAN patients [6]. However, due to the lack of large-scale studies, the limitation of animal models and current low-throughput genetic studies, the crucial genes involved in the development and effective treatment of IgAN have remained elusive.

Bioinformatics studies have been widely performed in various fields to extract potential information and reveal the underlying mechanics of various diseases. Recently, bioinformatics analysis has gradually provided insight into the molecular mechanisms of kidney disease. For example, PSMB8, as a novel hub gene, plays a significant role in the occurrence of membrane nephropathy [7]. In lupus, bioinformatics analysis revealed that CD38 and CCL2 are hub macrophage-related genes [8]. Additionally, EST1 may be a drug target for diabetic nephropathy treatment [9]. Currently, only a few bioinformatics analyses have been performed on IgAN; its critical associated genes and interactions have not been thoroughly investigated.

In the present study, two original microarray datasets were selected from the Gene Expression Omnibus (GEO) database. After identifying the differentially expressed genes (DEGs) in IgAN patients and control group, we employed the Database for Annotation, Visualization and Integration Discovery (DAVID) to identify the functions of the identified DEGs and performed Gene Ontology (GO) and Kyoto Encyclopedia of Genes and Genomes (KEGG) pathway analyses. The protein–protein interaction (PPI) network was generated using the STRING database, and hub genes and the most significant module among the PPI networks were identified using cytoHubba and the Molecular Complex Detection (MCODE) plug-in.

The present study aimed to identify potential novel candidate hub genes to diagnose and treat IgAN.

## Methods

### Microarray data

The microarray data were downloaded from the GEO database (http://www.ncbi.nlm.nih.gov/geo) using IgAN as the search term. GSE93798 is based on the Affymetrix Human GeneChip U133 2.0 platform (includes 42 samples, 20 IgAN patients and 22 healthy controls). GSE37460 is based on the Human Genome U133A Affymetrix platform (includes 54 samples, 27 IgAN patients and 27 healthy controls).

### Identification of DEGs

The DEGs were identified based on the series matrix file using the Limma package in R software (version 3.5.0). An adj *P* value < 0.01 and a |log FC (fold change) | ≥ 1 were defined as the thresholds for DEG screening. The DEGs overlapped between the two datasets were identified and then used for further functional enrichment analysis. The overlapped DEGs were subjected to bidirectional hierarchical clustering analysis using the Pheatmap package in R to recognize and visualize the differences in DEGs between IgAN and the control.

### Enrichment analysis of the DEGs

The DAVID (http://david.abcc.Ncifcrf.gov/) [10] tool was used to conduct GO/KEGG (http://www.genome.jp/kegg/pathway.html) [11] pathway enrichment analyses for DEGs. The number of enrichment genes (count number) ≥ 2 and *P* value < 0.05 were chosen as cut-off criteria.

### PPI network construction and module analysis hub gene identification

The DEGs identified were subjected to PPI analysis using the search functionality of STRING (http://string.embl.de/) [12] to explore the association between the DEGs, and a network interaction matrix was built. An interaction with a combined score > 0.4 was set as the cut-off value. Next, the network was visualized using Cytoscape software [13], which is a broadly used tool to visualize the interaction networks among numerous biomolecules, including proteins and genes. The MCODE plug-in was used to identify the most significant module in the PPI networks with MCODE scores > 5, degree cut-off = 2, node score cut-off = 0.2, max depth = 100 and k score = 2. CytoHubba [14] is a tool used to identify hub objects and subnetworks from a complex interactome. ‘MCC’ is a topological analysis method in CytoHubba that was used to identify featured nodes and the hub genes from all the DEGs. The biological processes of the hub genes were visualized using the Biological Networks Gene Ontology tool (BiNGO) (version 3.0.3) plug-in of Cytoscape [15], with a significance threshold of 0.01 and *Homo sapiens* as the selected organism.

### External microarray dataset validation

To validate the expression of FCER1G and ITGB2 in other glomerulonephritis types and IgAN, we used GSE104948 as an independent validate cohort, comprising focal segmental glomerular sclerosis(FSGS), minimal change disease (MCD), membranous nephropathy (MN), and thin membranous disease (TMD). The relative mRNA expression levels of FCER1G and ITGB2 in each patient were extracted from the raw data, which were then analyzed by Graphpad Prism 8. The data were presented as mean ± standard deviation. One-way analysis of variance was used to examine the differences between different groups. *P* < 0.05 was considered to indicate a statistically significant difference.

## Results

### Identification of DEGs

**Fig. 1:**
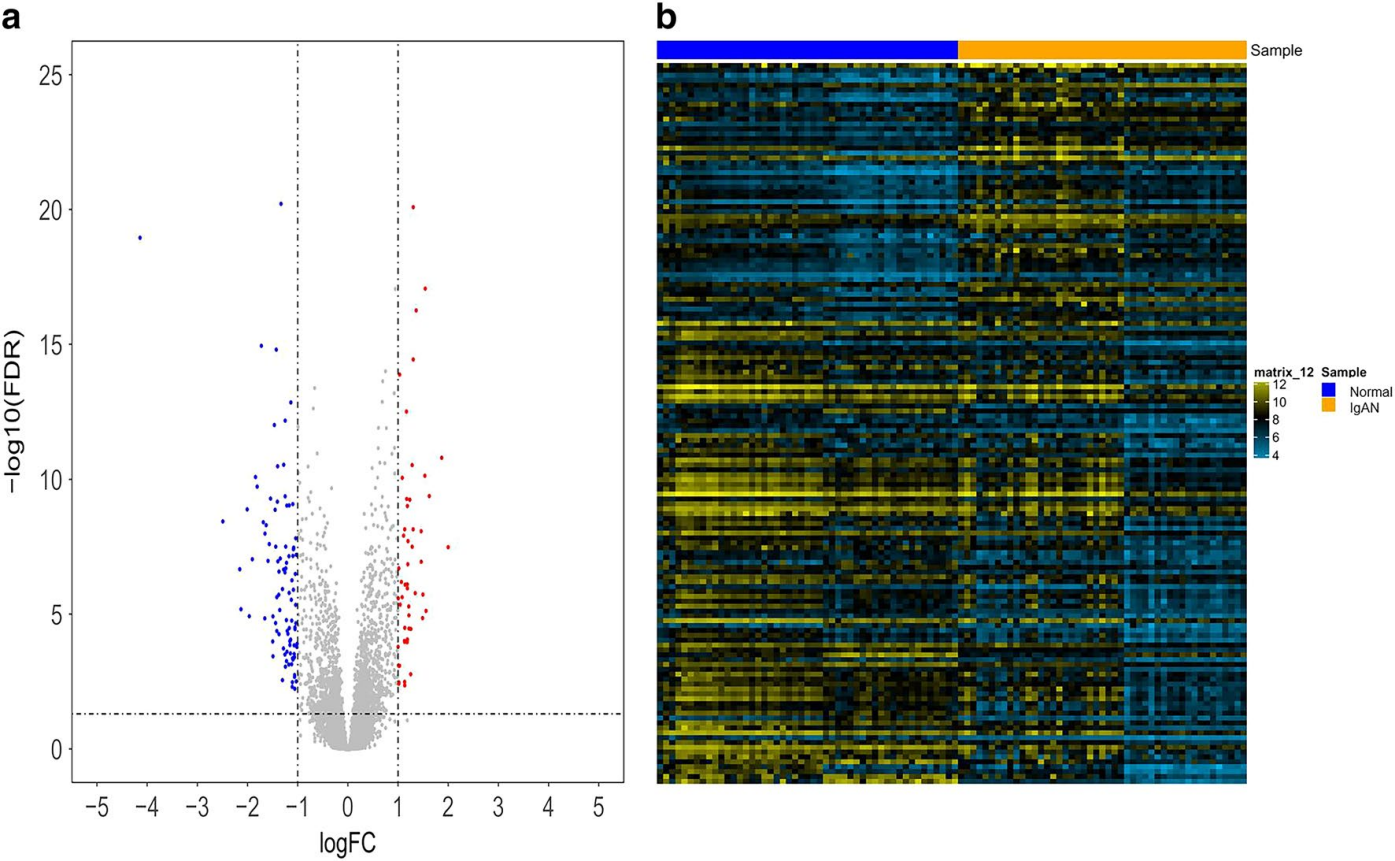
Identification and hierarchical clustering of DEGs. **a** Volcano plot of DEGs in IgAN. The cut-off criteria were |log2Fc| > 1 and *P* value < 0.05. The orange dots represent the upregulated genes, and the blue dots denote the downregulated genes. The gray dots indicate the genes with a |log2Fc| < 1 and/or *P* value > 0.05. **b** Heat map of the DEGs. Horizontal band with the cluster tree at the top: blue, normal samples; orange, IgAN. Each row represents a single gene. Blue, downregulated DEGs; orange, upregulated DEGs. The depth of the color denotes the change degree

We identified 96 samples, comprising 47 IgAN samples and 49 normal samples, in the two datasets. Based on the cut-off criteria of |log2 FC| ≥ 1.0 and adj *P*-value ≤ 0.05, 148 overlapping DEGs were shown by a Venn diagram obtained from the IgAN group vs. control group, comprising 53 upregulated and 95 downregulated genes. The results of the expression level analysis are presented in a volcano plot in Fig. 1a. As indicated in the clustering heat map (Fig. 1b), these DEGs could well distinguish the IgAN and control group completely.

### Gene ontology and KEGG analyses of DEGs

**Fig. 2:**
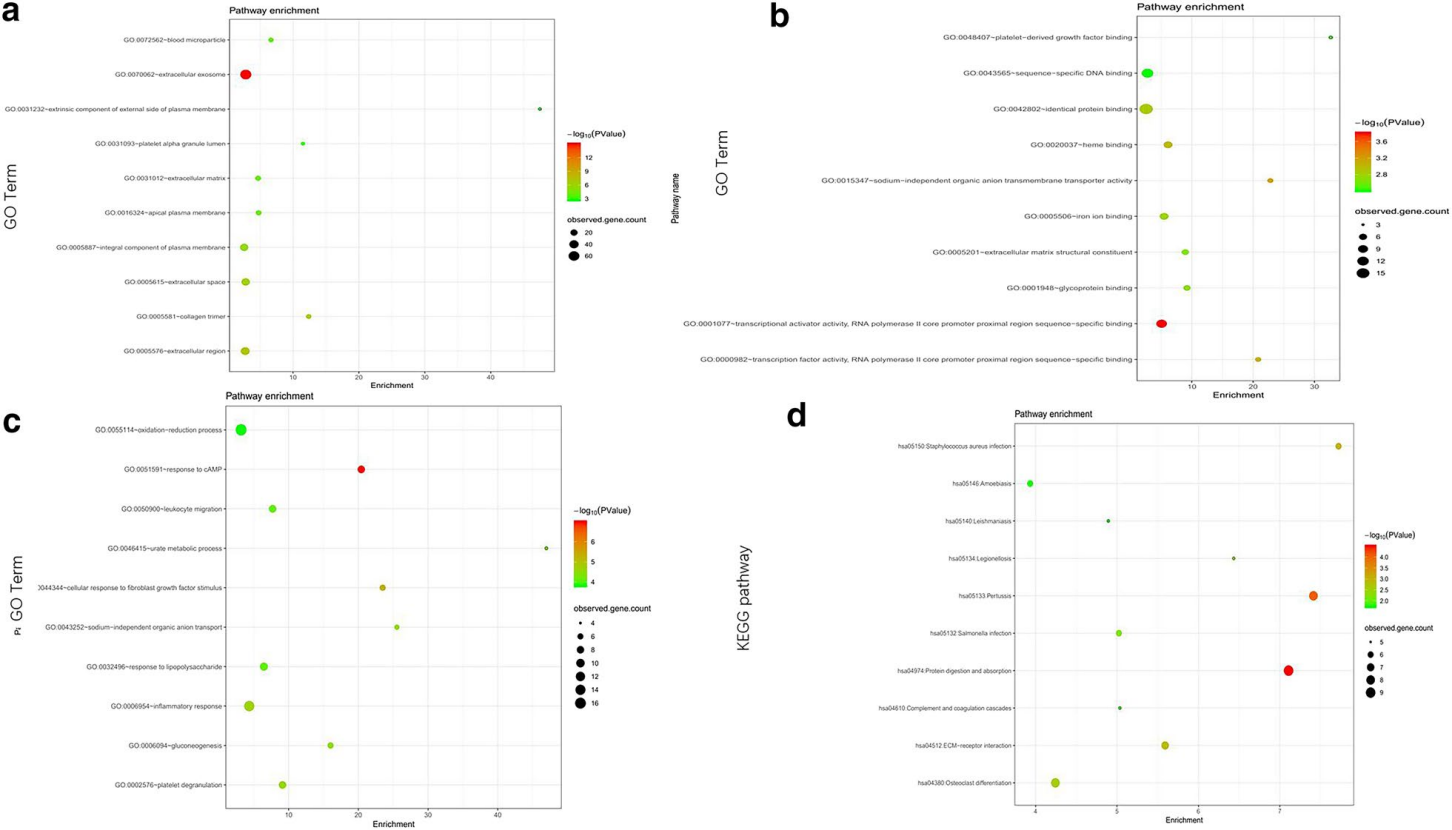
GO and KEGG pathway enrichment analysis of DEGs. The color depth of the nodes refers to the *P*-value. The size of the nodes refers to the number of genes. **a** GO CC terms. **b** GO MF terms. **c** GO BP terms. **d** KEGG pathway of DEGs

**Fig. 3:**
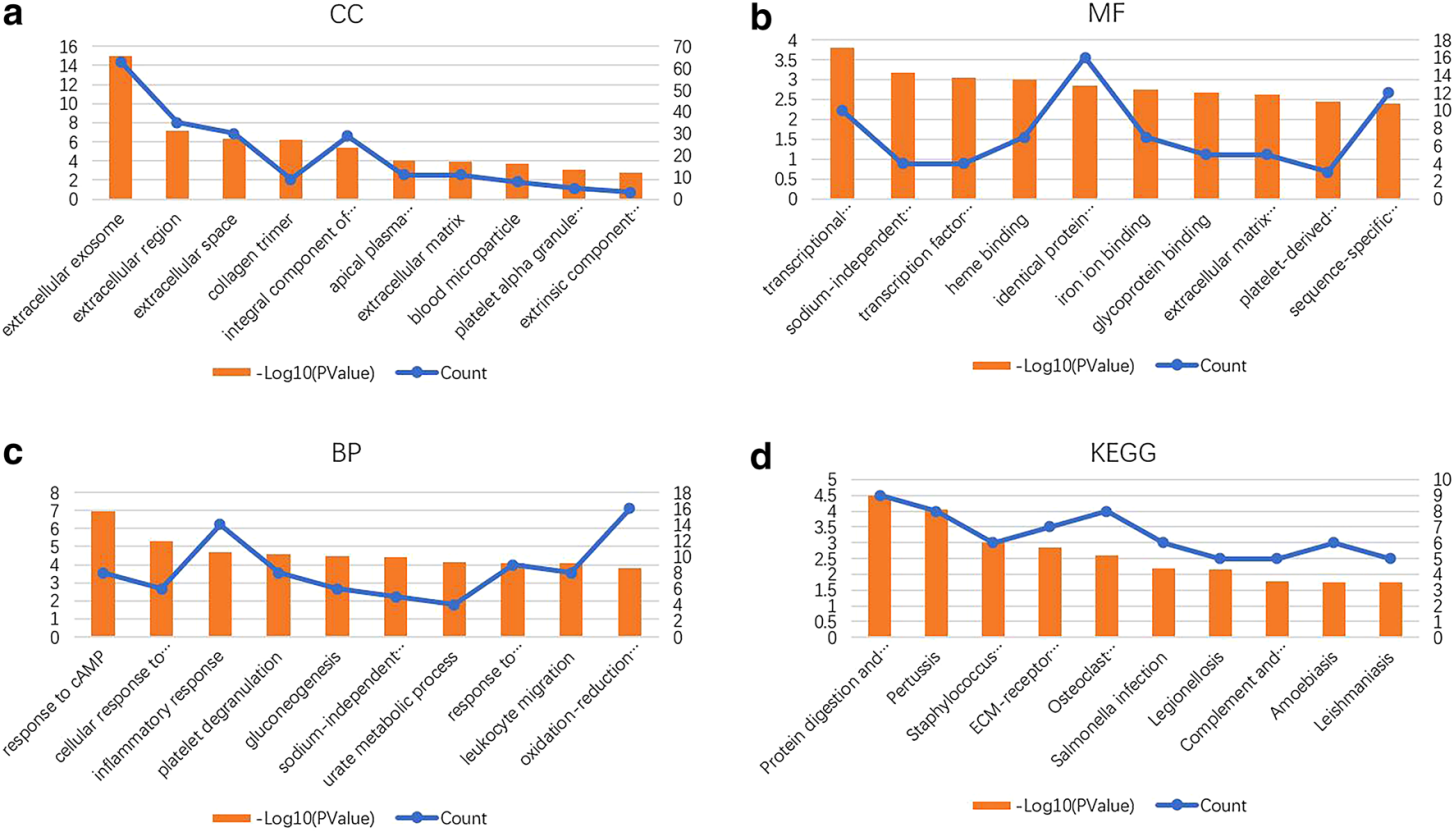
GO and KEGG pathway enrichment analysis of DEGs. **a** GO CC terms. **b** GO MF terms. **c** GO BP terms. **d** KEGG pathway of DEGs

To investigate the biological classification of DEGs, the overall genes in three ontologies were identified using DAVID. The cut-off criterion was set as *P* < 0.05. The GO function annotation is divided into three functional groups, cell component (CC), molecular function (MF), and biological process (BP). The CC terms of the DEGs were significantly enriched in extracellular exosome, region and space (Figs. 2a, 3a). The MF terms were mainly enriched in transcriptional activator activity and RNA polymerase II core promoter proximal region sequence-specific binding and heme binding identical protein binding (Figs. 2b, 3b). The changes in BP were significantly enriched in the response to cAMP, cellular response to fibroblast growth factor stimulus and inflammatory response (Figs. 2c, 3c).

KEGG pathway enrichment analysis revealed that the DEGs were mainly enriched in protein digestion and absorption, Pertussis and *Staphylococcus aureus* infection (Figs. 2d, 3d).

### Construction of the PPI network and module analysis

**Fig. 4:**
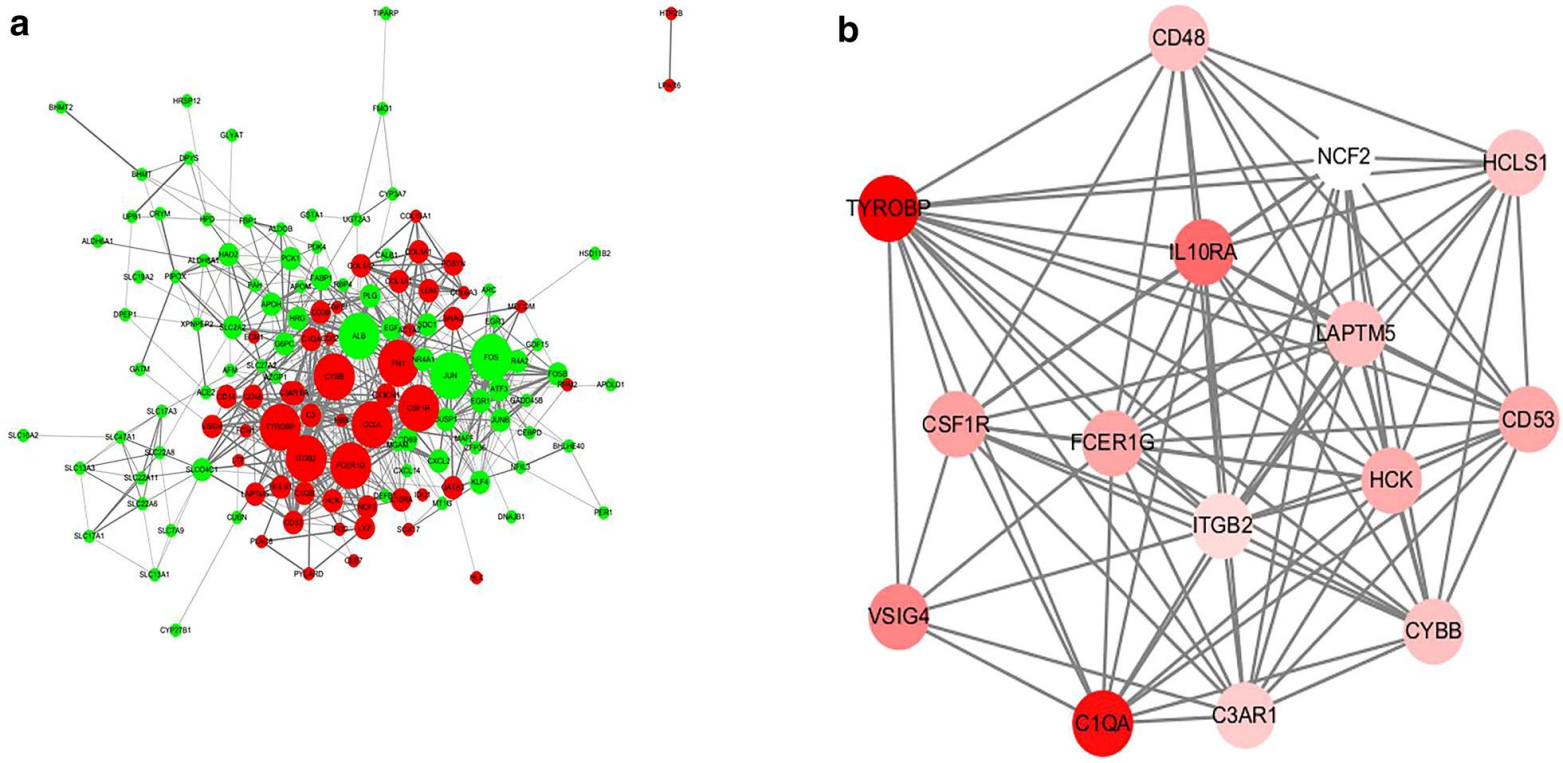
PPI network and the most significant module of DEGs. **a** The PPI network of DEGs was constructed using Cytoscape. Green, downregulated DEGs; red, upregulated DEGs. The size of the nodes refers to the gene degree. **b** The most significant module was obtained from the PPI network. The depth of the color denotes the change degree

To further investigate the interaction among the 148 DEGs, a PPI network was constructed from STRING (Fig. 4a). The most significant module was obtained using Cytoscape MCODE plug-in. The module comprised 15 nodes and 89 edges, including the Fc fragment of IgE receptor Ig (FCER1G), HCK proto-oncogene, Src family tyrosine kinase (HCK), TYRO protein tyrosine kinase binding protein (TYROBP), V-set and immunoglobulin domain containing 4 (VSIG4), colony stimulating factor 1 receptor (CSF1R), complement C1q A chain (C1QA), complement C3a receptor 1 (C3AR1), cytochrome b-245 beta chain (CYBB), hematopoietic cell-specific Lyn substrate 1 (HCLS1), integrin subunit beta 2 (ITGB2), interleukin 10 receptor subunit alpha (IL10RA), lysosomal protein transmembrane 5 (LAPTM5), neutrophil cytosolic factor 2 (NCF2), CD48 and CD53, which exhibited the highest score, 12.714 (Fig. 4b). These findings indicate that these genes exhibited higher hub degrees and could play critical roles in the development of IgAN.

**Fig. 5:**
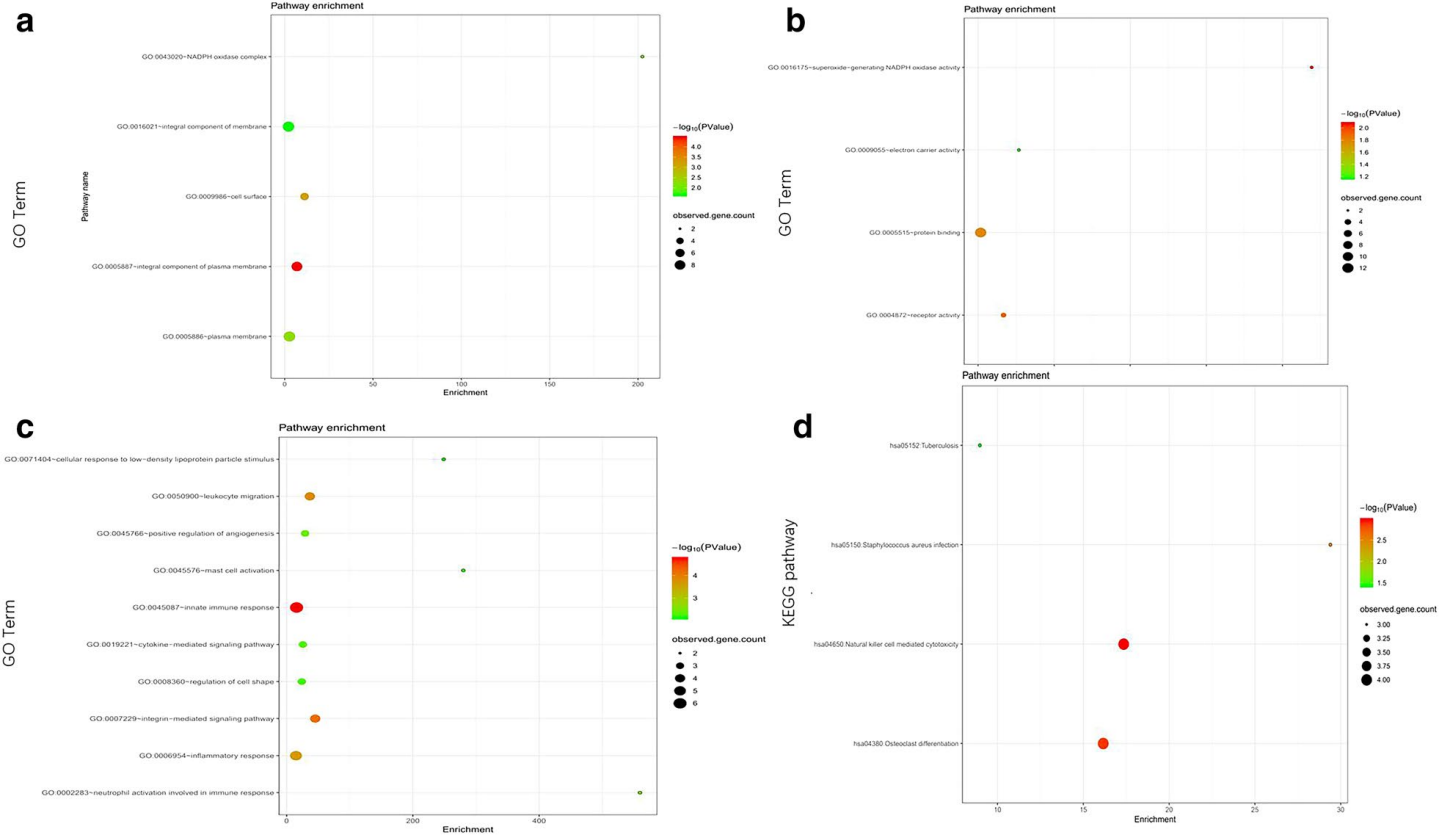
GO pathway enrichment analysis of DEGs in the most significant module. GO pathway enrichment analysis of DEGs in the most significant module obtained from the PPI network. The color depth of the nodes refers to the *P*-value. The size of the nodes refers to the number of genes. **a** GO CC terms. **b** GO MF terms. **c** GO BP terms. **d** KEGG pathway of DEGs

**Fig. 6:**
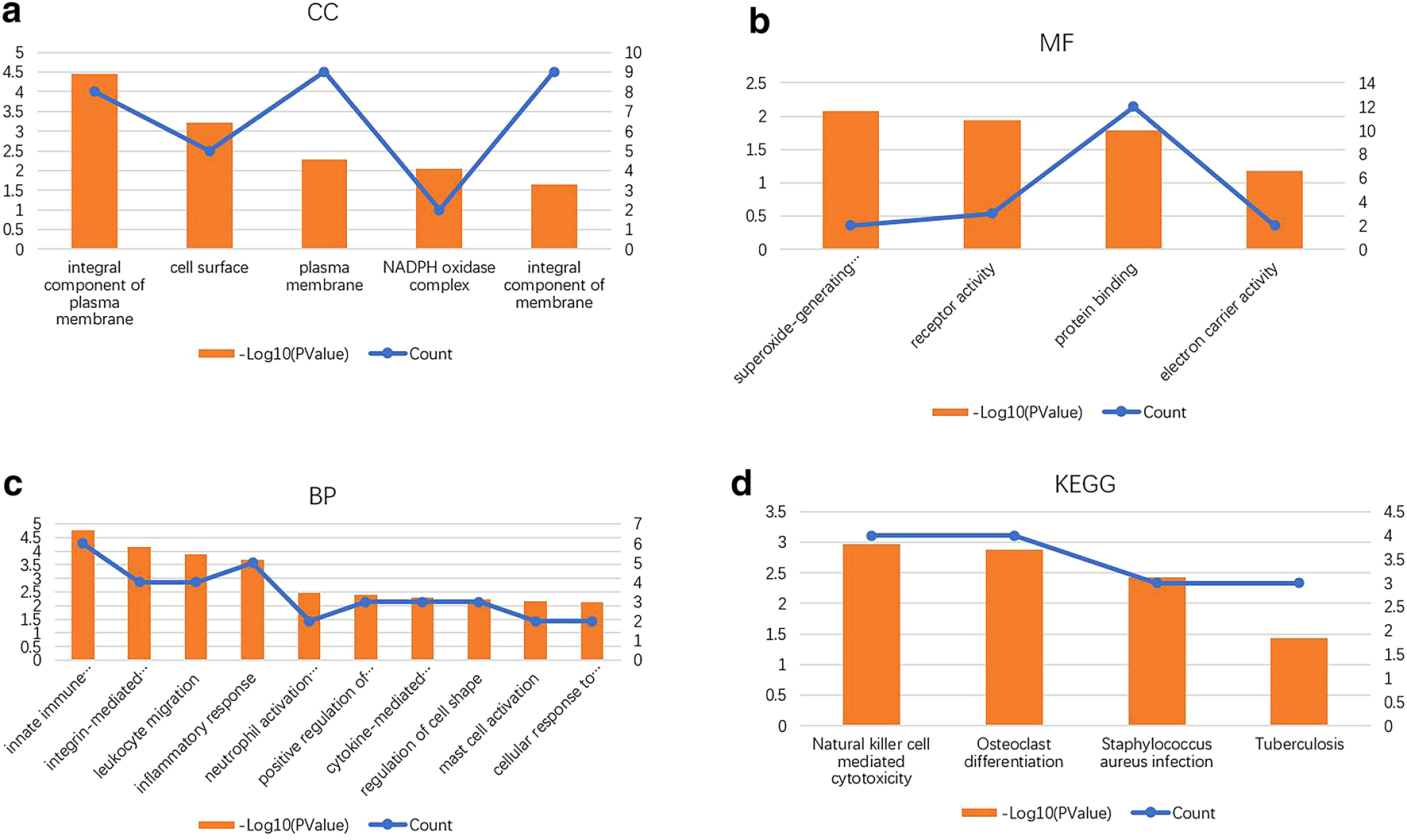
GO pathway enrichment analysis of DEGs in the most significant module. **a** GO CC terms. **b** GO MF terms. **c** GO BP terms. **d** KEGG pathway of DEGs

GO analysis of the module showed that the CC terms of the DEGs were mostly enriched in integral component of plasma membrane, cell surface and plasma membrane (Figs. 5a, 6a). The MF terms were mainly enriched in superoxide-generating NADPH oxidase, receptor activity and protein binding (Figs. 5b, 6b). The BP terms were mainly enriched in the innate immune response, integrin-mediated signaling pathway and inflammatory response (Figs. 5c, 6c).

KEGG pathway enrichment analysis revealed that the DEGs were mainly enriched in tuberculosis, natural killer cell-mediated cytotoxicity, osteoclast differentiation and *Staphylococcus aureus* infection (Figs. 5d, 6d).

### Identification and analysis of hub genes

**Fig. 7:**
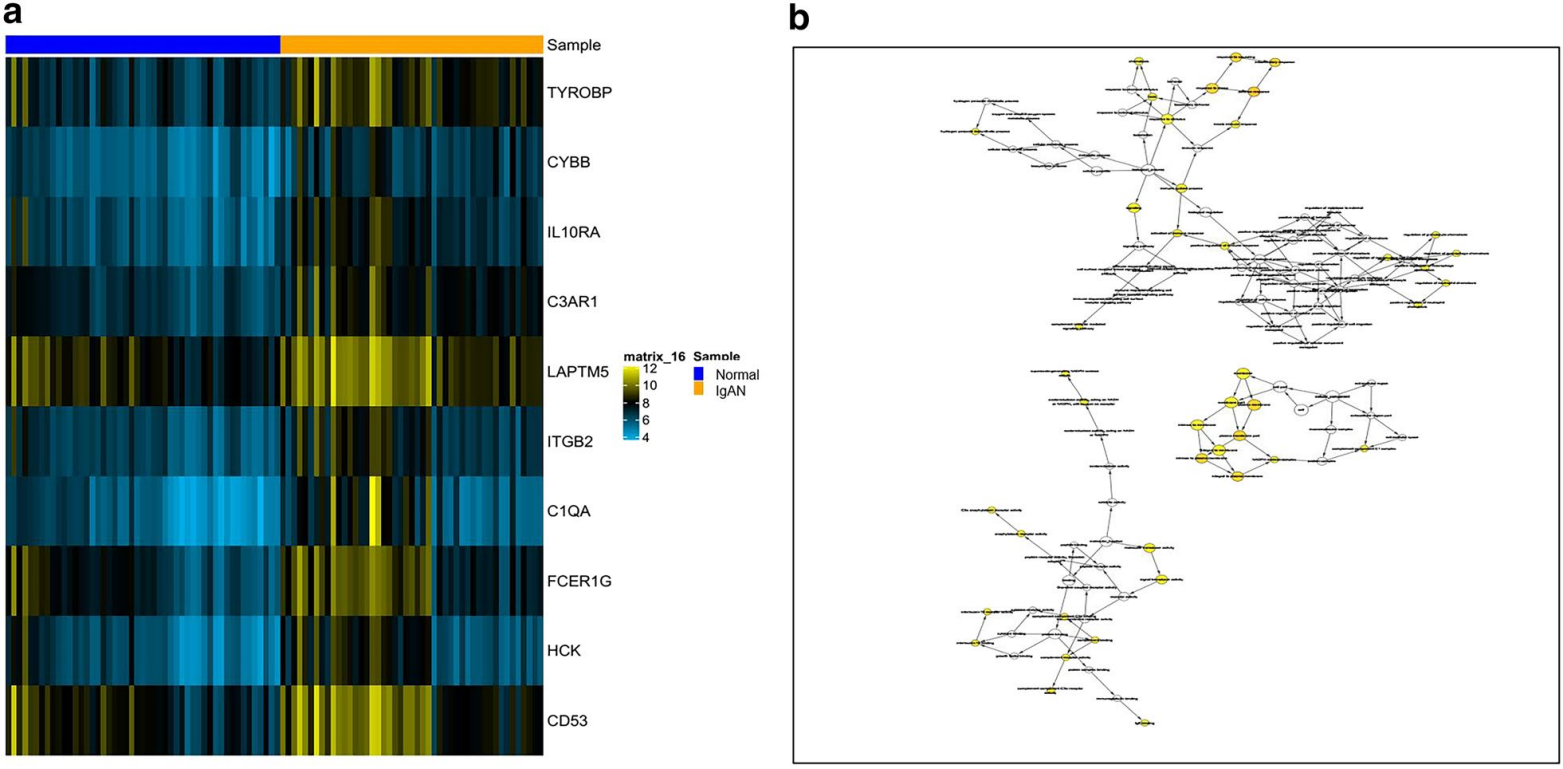
Interaction network and biological process analysis of the hub genes. **a** Heat map of the hub genes. Horizontal band with the cluster tree at the top: blue, normal samples; orange, IgAN. Each row represents a single gene. Blue, downregulated DEGs; orange, upregulated DEGs. The depth of the color denotes the change degree. **b** GO enrichment of hub genes was analyzed using BiNGO

We exported the STRING data to Cytoscape to construct and visualize the PPI network by implementing cytoHubba. Thereafter, we implemented the MCC method to evaluate the significance of the genes in the network. The top ten genes included IL10RA, ITGB2, HCK, C3AR1, CYBB, LAPTM5, FCER1G, CD53, C1QA and TYROBP. Hierarchical clustering of the hub genes was performed as indicated in the clustering heat map (Fig. 7a); these hub genes could well distinguish the IgAN and control group completely. The biological process analysis of hub genes was performed using the BiNGO plug-in shown in Fig. 7b.

**Fig. 8:**
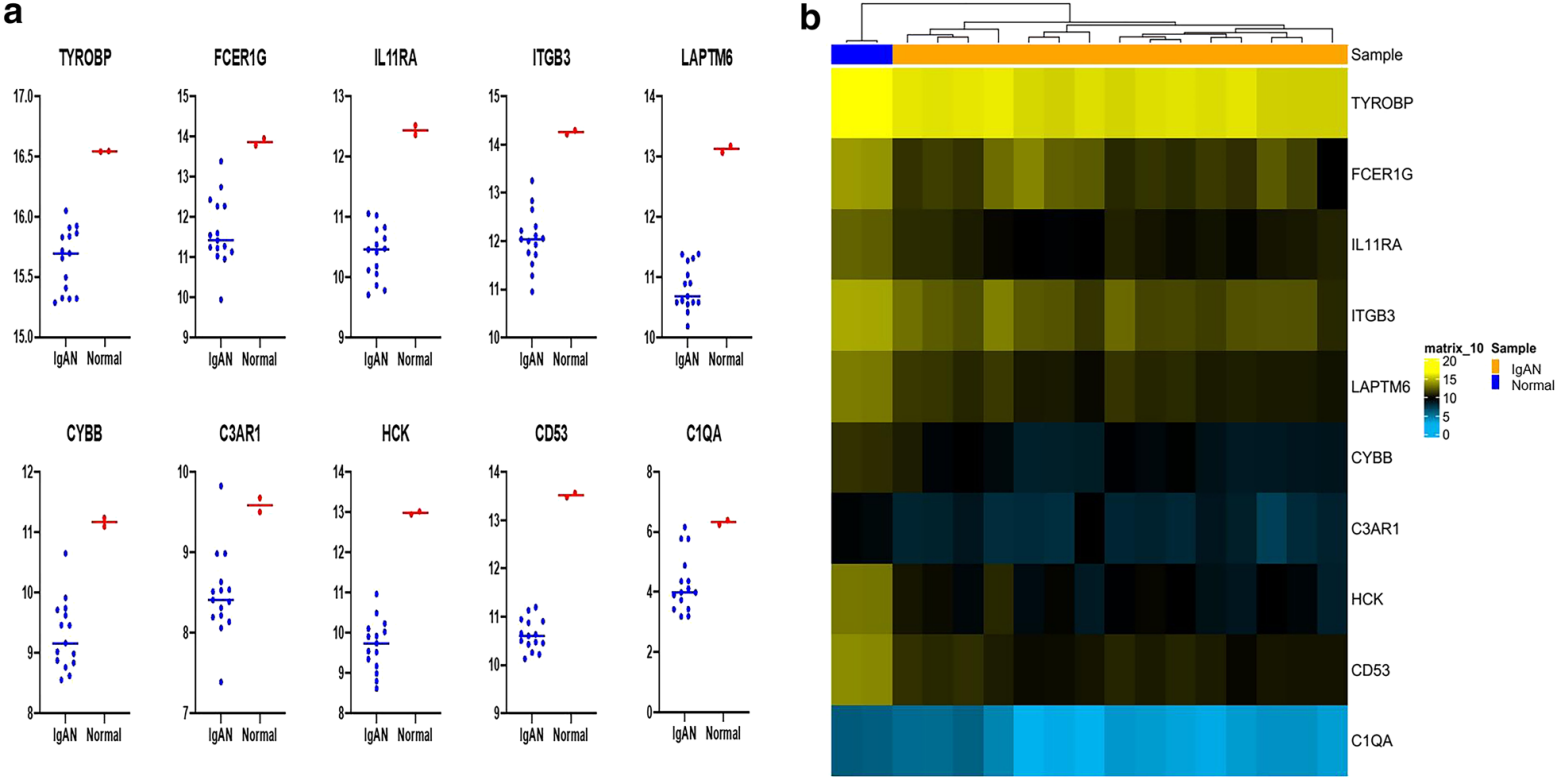
Hub genes analysis in blood monocytes sample. **a** Scatter plot of hub genes. **b** Heat map of the hub genes in the blood monocyte sample. Horizontal band with the cluster tree at the top: blue, normal samples; orange, IgAN. Each row represents a single gene. Blue, downregulated DEGs; orange, upregulated DEGs. The depth of the color denotes the change degree

For further analysis, the microarray dataset GSE58539 was downloaded from GEO. This dataset contained 17 monocyte samples, including 15 monocytes samples isolated from IgAN patients and 2 monocytes samples isolated from a healthy control group. We used these selected hub genes for analysis. The scatter plot showed that each hub gene was significantly different between the IgAN and control group (Fig. 8a). Hierarchical clustering of the hub genes was performed. As indicated in the clustering heat map (Fig. 8b), the hub genes could well distinguish the IgAN and control group in monocyte sample.

**Fig. 9:**
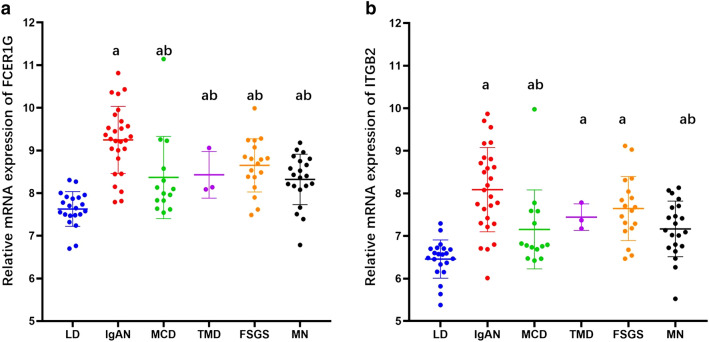
Validation of FCER1G and ITGB2 gene expression in IgAN and other primary glomerulonephritis types. **a** Scatter plot of FCER1G genes in IgAN and other primary glomerulonephritis types. **b** Scatter plot of ITGB2 genes in IgAN and other primary glomerulonephritis types. *a* compared with LD *P* <0.05, *b* compared with IgAN *P* <0.05

FCER1G and ITGB2 are the first and second-ranked hub genes. We further validated their gene expression in IgAN and determined whether they are specific for IgAN. We validated the relative gene expression in another independent cohort that combined IgAN and other primary glomerulonephritis types (such as FSGS, MCD, MN, and TMD) from the GEO database (GSE104948). Compared with LD, FCER1G and ITGB2 overexpression was observed in all the disease groups (*P* < 0.05) while the IgAN group showed much higher expression than other diseases (Fig. 9). Compared with the other glomerulonephritis types, FCER1G expression was much higher in IgAN (*P* < 0.05). ITGB2 expression has obvious overexpression in IgAN compared with the MN and MCD groups (*P* < 0.05).

## Discussion

Bioinformatics analysis plays an important role in disease studies, facilitating the understanding of pathogenesis by integrating data at the genome level with systematic bioinformatics methods. In the present study, 148 DEGs were identified from microarray data by reanalyzing the datasets that could distinguish between the IgAN and healthy controls. Previous studies have shown a significant association of IgAN development and prognosis with the inflammatory reaction, and activation of TGF-β signaling is closely related to fibrosis in IgAN [16, 17]. In our study, enrichment analysis revealed that the DEGs were significantly enriched in response to cAMP, cellular response to fibroblast growth factor stimulus and inflammatory response, a finding that is consistent with that in a previous study.

Infection plays an important role in the onset of IgAN, and nearly 30% of patients have a clear history of disease exacerbation after upper respiratory or gastrointestinal infections. Novak et al. reported that viruses (e.g., Epstein–Barr virus) or bacteria (e.g., *Streptococcus*) expressing GalNAc-containing moieties induce the development of IgG antiglycan autoantibodies, which might subsequently cross-react with glycans on IgA1, resulting in the formation of IgA1–IgG complexes [18]. This ‘molecular mimicry’ could also explain the association of macroscopic hematuria with upper respiratory tract infections. Yamamoto Y reported that the antigens of *Haemophilus parainfluenzae* are detected in the renal tissue of patients with IgAN [19]. In our study, KEGG analysis revealed that the DEGs were mainly enriched in pertussis and *Staphylococcus aureus* infection, a finding that coincides with the above studies.

Using STRING and MCODE, we selected the most important module, which comprised 15 nodes and 89 edges, including CSF1R, IL10RA, ITGB2, HCK, NCF2, C3AR1, CYBB, HCLS1, CD48, C1QA, VSIG4, LAPTM5, FCER1G, CD53 and TYROBP. Further GO analysis revealed that the BP was mainly enriched in the innate immune response, integrin-mediated signaling pathway and inflammatory response. Previous studies have shown a significant association of IgAN development and prognosis with the inflammatory reaction and innate immune response. Toll-like receptors (TLRs) are the key components of the mammalian innate immune system and mediate immune and inflammatory responses through binding PAMPs and/or DAMPs [20]. Many studies have confirmed the elevated expression of TLR4 mRNA in IgAN rats. In an in vitro coculture system of IgA and mesangial cells, TLR4 mediates MAPK activation and MCP-1 secretion, indicating that TLR4 is engaged in glomerular mesangium damage by inducing inflammatory cytokines in IgAN [21]. TLR4 is also involved in the activation of NF-κB, triggering the transcription of mRNA encoding many inflammatory mediators, such as cytokines, chemokines, and fibrinogen and contributing significantly to the effects of the innate and adaptive immune responses [22].

We further implemented the MCC method and selected 10 hub genes, all of which overlapped with the important module selected by MCODE. Hierarchical clustering of the hub genes showed that these hub genes could well distinguish the IgAN and control groups completely. We further introduced these genes into blood samples for testing, and the results showed that the genes also played a crucial role in differentiating disease from control in the blood tissue.

Hck is a member of the highly conserved Src family of cytoplasmic protein tyrosine kinases that transduce various extracellular signals. Hck has been reported to be significantly upregulated in diabetic nephropathy, IgA nephropathy, and lupus nephritis, and is a key mediator of renal fibrosis via its effects on inflammation, fibroblast cell proliferation, and regulation of TGF-β signaling [23].

LAPTM5, which is preferentially expressed in hematopoietic cells and localized to the lysosome, was initially isolated by a subtractive hybridization strategy between hematopoietic and nonhematopoietic cells. A recent study showed that LAPTM5 is a positive regulator of proinflammatory signaling pathways by facilitating NF-κB and MAPK signaling, as well as proinflammatory cytokine production in macrophages [24, 25]. CYBB is also responsive to several inflammatory cytokines such as IFN-γ, LPS, and TNF-α [26]. CD53 codes for cluster of differentiation 53, a leukocyte surface antigen. Many studies have indicated that CD53 plays a substantial role in cellular stability and the inflammatory response to adverse conditions [27]. The inflammatory response plays an important role in IgAN, and the above hub genes were all identified to be involved in the pathogenesis of IgAN.

FCER1G is a protein coding gene that interacts with other factors and participates in various nuclear pathways [28]. Specifically, FCER1G is a constitutive component of the high-affinity immunoglobulin E receptor and interleukin-3 receptor complex and is mainly involved in mediating the allergic inflammatory signaling of mast cells, selectively mediating the production of interleukin 4 by basophils, and initiating the transfer from T cells to the effector T-helper 2 subset [29]. Additionally, FCER1G is associated with the progression of clear-cell renal cell carcinoma and may improve prognosis by affecting immune-related pathways. Furthermore, FCER1G is a critical molecule in signaling pathways and is widely involved in various immune responses and cell types [30]. Until now, no study has reported the association of FCER1G with IgAN.

ITGB2 is a protein coding gene that encodes an integrin beta chain, which combines with multiple different alpha chains to form different integrin heterodimers. Integrins are integral cell-surface proteins that participate in cell adhesion as well as cell surface-mediated signaling. The encoded protein plays an important role in the immune response, and defects in this gene cause leukocyte adhesion deficiency. ITGB2 was reported to be involved in cellular adhesion and ECM remodeling in patients with renal cancer [31]. Furthermore, ITGB2 was identified to be closely associated with apoptosis in patients with Alzheimer’s disease [32]. Bioinformatics analysis in CKD patients showed that ITGB2, CTSS and CCL5 are correlated negatively with the eGFR of CKD patients [33].

In our study, FCER1G and ITGB2 were the first- and second-ranked hub genes, respectively, and BiNGO analysis confirmed that FCER1G is directly involved in the innate immune response. Further analysis uncovered that, except for IgAN, both hub genes exhibit higher expression in other primary glomerulonephritis types (FSGS, MCD, MN TMD). The latter finding indicates that these hub genes may also be associated with the pathogenesis of other primary glomerulonephritis types. Although these two genes have the highest expression in IgAN compared with other primary glomerulonephritis types, they may play an important role in IgAN but are not specific for the disease. Presently, limited research has reported the association between ITGB2 or FCER1G and IgAN. Further investigation of these two genes is warranted.

## Conclusions

Through bioinformatics analysis, we identified hub genes involved in the pathological changes of IgAN. These genes not only can be used in tissue samples, but also play important roles in blood samples. The present study is the first to apply an integrated bioinformatics analysis to investigate novel candidate genes and mechanisms involved in the pathogenesis of IgAN. Among the genes, ITGB2 and FCER1G may play important roles in the development of IgAN and act as potential candidate molecular targets that deserve further research.

## Data Availability

All data of the databases are available.

## Abbreviations

IgAN: Immunoglobulin A nephropathy
DEGs: Differentially expressed genes
PPI: Protein–protein interaction
BiNGO: Biological Networks Gene Ontology
GO: Gene Ontology
Gd-IgA1: Galactose-deficient IgA1
GEO: Gene Expression Omnibus
DAVID: Database for Annotation, Visualization and Integration Discovery
KEGG: Kyoto Encyclopedia of Genes and Genomes
MCODE: Molecular Complex Detection
CC: Cell component
MF: Molecular function
BP: Biological process
TLRs: Toll-like receptors
LAPTM5: Lysosomal-associated multi-spanning membrane protein 5
FCER1G: Fc fragment of IgE receptor Ig
ITGB2: Integrin subunit beta 2
TYROBP: TYRO protein tyrosine kinase binding protein
C1QA: Complement C1q A chain
C3AR1: Complement C3a receptor 1
CYBB: Cytochrome b-245 beta chain
HCK: HCK proto-oncogene, Src family tyrosine kinase
IL10RA: Interleukin 10 receptor subunit alpha
VSIG4: V-set and immunoglobulin domain containing 4
CSF1R: Colony stimulating factor 1 receptor
HCLS1: Hematopoietic cell-specific Lyn substrate 1
NCF2: Neutrophil cytosolic factor 2
TMD: Thin membranous disease
FSGS: Focal segmental glomerular sclerosis
MCD: Minimal change disease
MN: Membranous nephropathy

## References

[1] Berger J, Hinglais N. Intercapillary deposits of IgA-IgG. J Urol Nephrol. 1968;74:694–5. 4180586

[2] Suzuki H, Kiryluk K, Novak J. The pathophysiology of IgA nephropathy. J Am Soc Nephrol. 2011;22:1795–803. 21949093 10.1681/ASN.2011050464 PMC3892742

[3] Zhu L, Zhang H. The genetics of IgA nephropathy: an overview from China. Kidney Dis. 2015;1:27–32. 10.1159/000381740 PMC4934795 27536662

[4] Serino G, Sallustio F, Cox SN, Pesce F, Schena FP. Abnormal miR-148b expression promotes aberrant glycosylation of IgA1 in IgA nephropathy. J Am Soc Nephrol. 2012;23:814–24. 22362909 10.1681/ASN.2011060567 PMC3338289

[5] Serino G, Sallustio F, Curci C, et al. Role of let-7b in the regulation of *N*-acetylgalactosaminyl transferase 2 in IgA nephropathy. Nephrol Dial Transplant. 2015;3:1132–9. 10.1093/ndt/gfv032 25744272

[6] Wang G, Kwan BC, Lai FM, et al. Urinary miR-21, miR-29, and miR-93: novel biomarkers of fibrosis. Am J Nephrol. 2012;36:412–8. 23108026 10.1159/000343452

[7] Tang WX, Wang Z, Cao YL, et al. Bioinformatic analysis reveals novel immune-associated hub genes in human membranous nephropathy. Genet Test Mol Biomarkers. 2019;23(1):23–31. 30526079 10.1089/gtmb.2018.0137

[8] Shu B, Fang Y, He W, Yang J, et al. Identification of macrophage-related candidate genes in lupus nephritis using bioinformatics analysis. Cell Signal. 2018;46:43–51. 29458096 10.1016/j.cellsig.2018.02.006

[9] Geng XD, Wang WW, Feng Z, et al. Identification of key genes and pathways in diabetic nephropathy by bioinformatics analysis. J Diabetes Investig. 2019;10(4):972–84. 30536626 10.1111/jdi.12986 PMC6626994

[10] Huang DW, Sherman BT, Lempicki RA. Systematic and integrative analysis of large gene lists using DAVID bioinformatics resources. Nat Protoc. 2009;4(1):44. 19131956 10.1038/nprot.2008.211

[11] Kanehisa M, Goto S. KEGG: Kyoto encyclopedia of genes and genomes. Nucleic Acids Res. 2000;28(1):27–30. 10592173 10.1093/nar/28.1.27 PMC102409

[12] Szklarczyk D, Franceschini A, Kuhn M, et al. The STRING database in 2011: functional interaction networks of proteins, globally integrated and scored. Nucleic Acids Res. 2011;39(suppl_1):561–8. 10.1093/nar/gkq973 PMC3013807 21045058

[13] Smoot ME, Ono K, Ruscheinski J, et al. Cytoscape 2.8: new features for data integration and network visualization. Bioinformatics. 2011;27(3):431–2. 21149340 10.1093/bioinformatics/btq675 PMC3031041

[14] Chin CH, Chen SH, Wu HH, et al. cytoHubba: Identifying hub objects and sub-networks from complex interactome. BMC Syst Biol. 2014;8(Suppl 4):S11. 25521941 10.1186/1752-0509-8-S4-S11 PMC4290687

[15] Maere S, Heymans K, Kuiper M. BiNGO: a Cytoscape plugin to assess over-representation of gene ontology categories in biological networks. Bioinformatics. 2005;21(16):3448–9. 15972284 10.1093/bioinformatics/bti551

[16] Hennino MF, Buob D, Van der Hauwaert C. miR-21-5p renal expression is associated with fibrosis and renal survival in patients with IgA nephropathy. Sci Rep. 2016;6:27209. 27264483 10.1038/srep27209 PMC4893709

[17] Tanaka K, Sugiyama H, Yamanari T, et al. Renal expression of trefoil factor 3 mRNA in association with tubulointerstitial fibrosis in IgA nephropathy. Nephrology. 2018;23:855–62. 29987860 10.1111/nep.13444 PMC6174951

[18] Novak J, Julian BA, Tomana M, et al. IgA glycosylation and IgA immune complexes in the pathogenesis of IgA nephropathy. Semin Nephrol. 2008;28:78–85. 18222349 10.1016/j.semnephrol.2007.10.009 PMC2241661

[19] Yamamoto Y, Hiki Y, Nakai NS, et al. Comparison of effective impact among tonsillectomy alone, tonsillectomy combined with oral steroid and with steroid pulse therapy on long-term outcome of immunoglobulin A nephropathy. Clin Exp Nephrol. 2013;17:218–24. 22926695 10.1007/s10157-012-0679-2 PMC3627042

[20] Takeda K, Kaisho T, Akira S. Toll-like receptors. Annu Rev Immunol. 2003;21:335–76. 12524386 10.1146/annurev.immunol.21.120601.141126

[21] Lim BJ, Lee D, Hong SW, Jeong HJ. Toll-like receptor 4 signaling is involved in IgA-stimulated mesangial cell activation. Yonsei Med J. 2011;52:610–5. 21623603 10.3349/ymj.2011.52.4.610 PMC3104456

[22] Chen X, Peng S, Zeng H, et al. Toll-like receptor 4 is involved in a protective effect of rhein on immunoglobulin a nephropathy. Indian J Pharmacol. 2015;47:27–33. 25821307 10.4103/0253-7613.150319 PMC4375814

[23] Wei C, Li L, Menon MC, et al. Genomic analysis of Kidney allograft injury identifies hematopoietic cell kinase as a key driver of renal fibrosis. J Am Soc Nephrol. 2017;28(5):1385–93. 27927780 10.1681/ASN.2016020238 PMC5407716

[24] Adra CN, Zhu S, Ko JL, et al. LAPTM5: a novel lysosomal-associated multispanning membrane protein preferentially expressed in hematopoietic cells. Genomics. 1996;35(2):328–37. 8661146 10.1006/geno.1996.0364

[25] Glowacka WK, Alberts P, Ouchida R, et al. LAPTM5 protein is a positive regulator of proinflammatory signaling pathways in macrophages. J Biol Chem. 2012;287(33):27691–702. 22733818 10.1074/jbc.M112.355917 PMC3431655

[26] Nauseef WM, Borregaard N. Neutrophils at work. Nat Immunol. 2014;15:602–11. 24940954 10.1038/ni.2921

[27] Bos SD, Lakenberg N, van der Breggen R, et al. A genome wide linkage scan reveals CD53 as an important regulator of innate TNF-alpha levels. Eur J Hum Genet. 2010;18(8):953–9. 20407468 10.1038/ejhg.2010.52 PMC2987381

[28] Carter CJ. Interactions between the products of the Herpes simplex genome and Alzheimer’s disease susceptibility genes: relevance to pathological-signaling cascades. Neurochem Int. 2008;52:920–34. 18164103 10.1016/j.neuint.2007.11.003

[29] Sweet RA, Nickerson KM, Cullen JL, et al. B cell-extrinsic Myd88 and Fcer1g negatively regulate autoreactive and normal B cell immune responses. J Immunol. 2017;199:885–93. 28659358 10.4049/jimmunol.1600861 PMC5547912

[30] Liang Y, Zhao M, Liang G, et al. Construction of special reporter to detect DNA methylation regulatory activity in FCER1G gene promoter through patch-methylation. Zhong Nan Da Xue Xue Bao Yi Xue Ban. 2013;38:120–4. 23456064 10.3969/j.issn.1672-7347.2013.02.002

[31] Boguslawska J, Kedzierska H, Poplawski P, et al. Expression of genes involved in cellular adhesion and extracellular matrix remodeling correlates with poor survival of patients with renal cancer. J Urol. 2016;195:1892–902. 26631499 10.1016/j.juro.2015.11.050

[32] Mahanian M, Weitzman R, Hayden EY, Rosenthal MJ, et al. 1α,25-dihydroxyvitamin D3 and resolvin D1 retune the balance between amyloid-β phagocytosis and inflammation in Alzheimer’s disease patients. J Alzheimer’s Dis. 2013;34:155–70. 23186989 10.3233/JAD-121735 PMC4040018

[33] Wang WP, Shen JX, Qi CJ, et al. The key candidate genes in tubulointerstitial injury of chronic kidney diseases patients as determined by bioinformatic analysis. Cell Biochem Funct. 2020. 10.1002/cbf.3545. 32340064 10.1002/cbf.3545

