# Editorial Expression of Concern: Bioinformatics analysis reveals novel hub gene pathways associated with IgA nephropathy

**DOI:** 10.1186/s40001-024-02253-0

**Published:** 2025-01-13

**Authors:** Xue Jiang, Zhijie Xu, Yuanyuan Du, Hongyu Chen

**Affiliations:** 1 Department of Nephropathy, Hangzhou Hospital of Traditional Chinese Medicine, Hangzhou, 310012 Zhejiang China; 2 Department of Urology, The First Affiliated Hospital School of Medicine, Zhejiang University, Hangzhou, 310009 Zhejiang China

**Editorial Expression of Concern: Eur J Med Res (2020) 25:40** 10.1186/s40001-020-00441-2

The Editors-in-Chief would like to alert readers that concerns have been raised about the statistical analysis presented in this article. The authors did not response to repeated requests from the journal for source data. Readers are therefore advised to interpret results with caution.

The authors have not replied to correspondence from the Publisher.

